# A case report of relapsing organizing pneumonia in rheumatoid arthritis with literature review

**DOI:** 10.1097/MD.0000000000049547

**Published:** 2026-06-26

**Authors:** Fangfang Wang, Fengmei Ge, Xuebin Wang

**Affiliations:** a Department of Rheumatology, Binzhou Medical University Hospital, Binzhou, Shandong, China.

**Keywords:** case report, fever, organizing pneumonia, relapse, rheumatoid arthritis

## Abstract

**Rationale:**

Organizing pneumonia (OP) rarely occurs in patients with rheumatoid arthritis (RA). We report a case of concurrent onset of OP and RA, in which OP relapsed after 9 years, while RA remained in a state of low disease activity.

**Patient concerns:**

A 65-year-old woman presented with pain in multiple joints accompanied by recurrent fever. Chest computed tomography (CT) showed inflammation in the right lung. After treatment for the infection proved ineffective, a right lung biopsy confirmed OP. Despite 9 years of antirheumatic treatment, the patient experienced recurrent fever and cough. Chest CT revealed bilateral pneumonia.

**Diagnoses:**

The patient underwent fiberoptic bronchoscopy, and recurrence of OP was confirmed by a second lung biopsy.

**Interventions:**

Initial treatment consisted of methylprednisolone 40 mg administered once daily, followed by a gradual tapering regimen combined with iguratimod 25 mg twice daily.

**Outcomes:**

The patient’s fever and cough subsided, and chest CT showed significant improvement. The erythrocyte sedimentation rate and C-reactive protein levels were normal.

**Lessons:**

OP can occur simultaneously with RA and may recur. Lung pathology plays a crucial role in the diagnosis of these conditions.

## 1. Introduction

Rheumatoid arthritis (RA) is a chronic autoimmune disorder predominantly affecting middle-aged and elderly females. Interstitial pneumonia (IP), an extra-articular manifestation of RA, is not only a common complication but also a harbinger of a poor prognosis. IP can occur before, during, or after an RA diagnosis. Among the pathological types of IP associated with RA, usual interstitial pneumonia (UIP) and nonspecific interstitial pneumonia (NSIP) are the most frequently encountered, whereas organizing pneumonia (OP) is less common.^[1,2]^ Here, we present a case involving the simultaneous occurrence of OP and RA. After treatment, the patient’s condition improved significantly. However, OP recurred 9 years later, whereas RA remained at a relatively low level of disease activity.

## 2. Case report

### 2.1. Patient information

**Figure 1.**
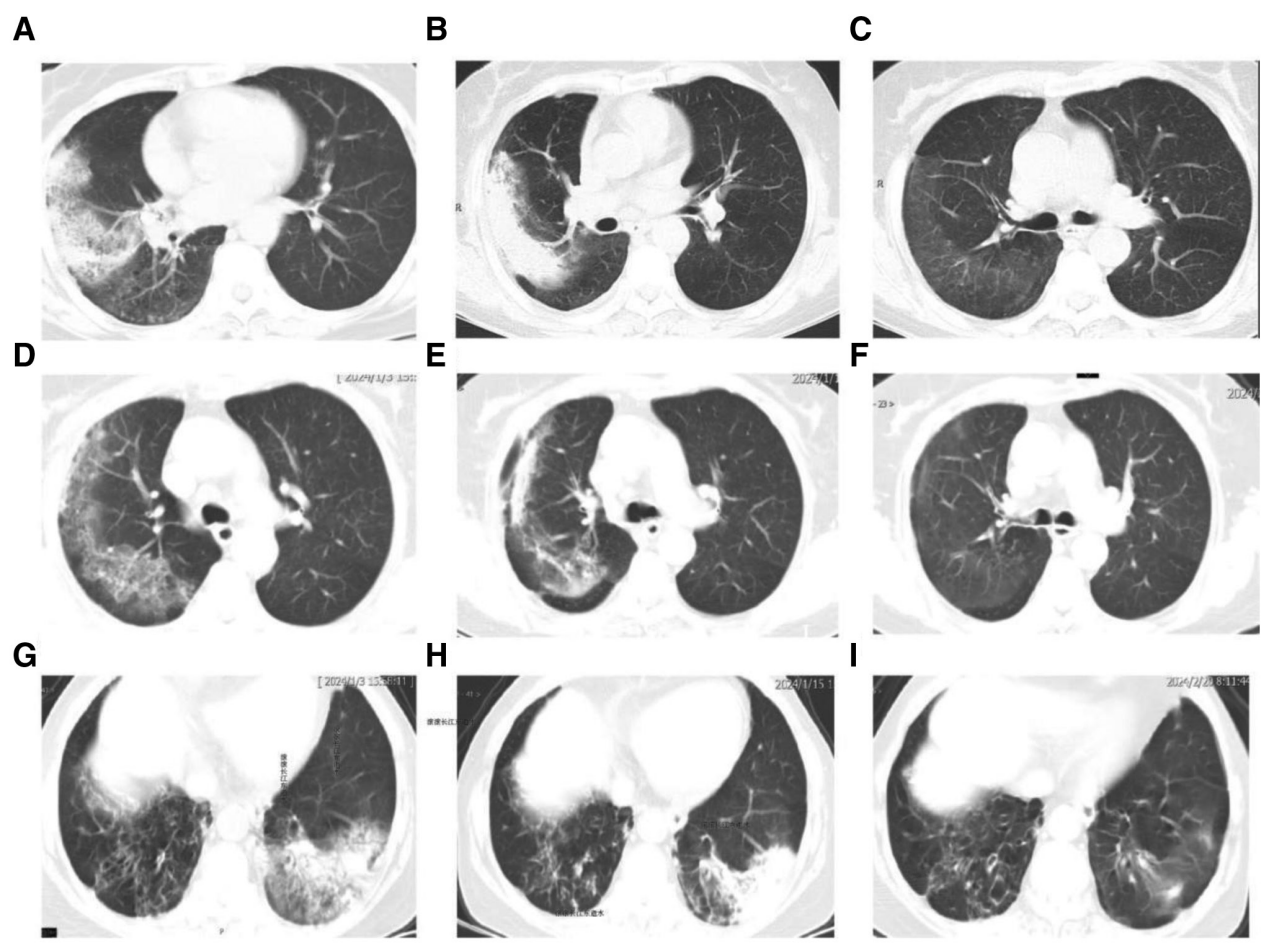
(A) Upon initial admission without prior treatment, chest CT demonstrated inflammation in the right lung. (B) Two weeks after anti-infective therapy, chest CT revealed no significant improvement in the right lung inflammation. (C) Three months following the initial diagnosis of organizing pneumonia and effective treatment, chest CT indicated that the right lung inflammation had largely resolved. (D, G) At the time of the second admission, without treatment in the interim, chest CT revealed inflammation in both the left and right lungs. (E, H) After 12 days of anti-infective treatment, chest CT showed no significant improvement in bilateral pneumonitis. (F, I) Fifty days after the initial diagnosis of OP and appropriate treatment, chest CT demonstrated that bilateral pneumonitis had largely subsided. CT = computed tomography.

A 65-year-old woman presented with symmetrical swelling and pain in multiple joints – including the metacarpophalangeal, wrist, and elbow joints – that had persisted for 1 month, accompanied by intermittent fever peaks of 39.3°C. The patient had a history of bronchiectasis. Laboratory tests revealed elevated levels of white blood cells (13.07 × 10^9^/L; 3.5–9.5 × 10^9^/L), neutrophils (10.76 × 10^9^/L; 1.8–6.3 × 10^9^/L), erythrocyte sedimentation rate (ESR) 78 mm/h (0–20 mm/h), C-reactive protein (CRP; 197 mg/L; 0–6 mg/L), rheumatoid factor (99 IU/mL; 0–20 IU/mL), and anti-CCP antibodies (16 U/mL; 0–5 U/mL). Antinuclear antibodies, HLA-B27, tumor markers, and markers of infection, including fungal, tuberculosis, and viral infection markers, as well as blood culture results, were negative. Chest computed tomography (CT) revealed right lung inflammation, localized interstitial changes, and bronchiectasis of the right lower lobe (Fig. 1A).

**Figure 2.**
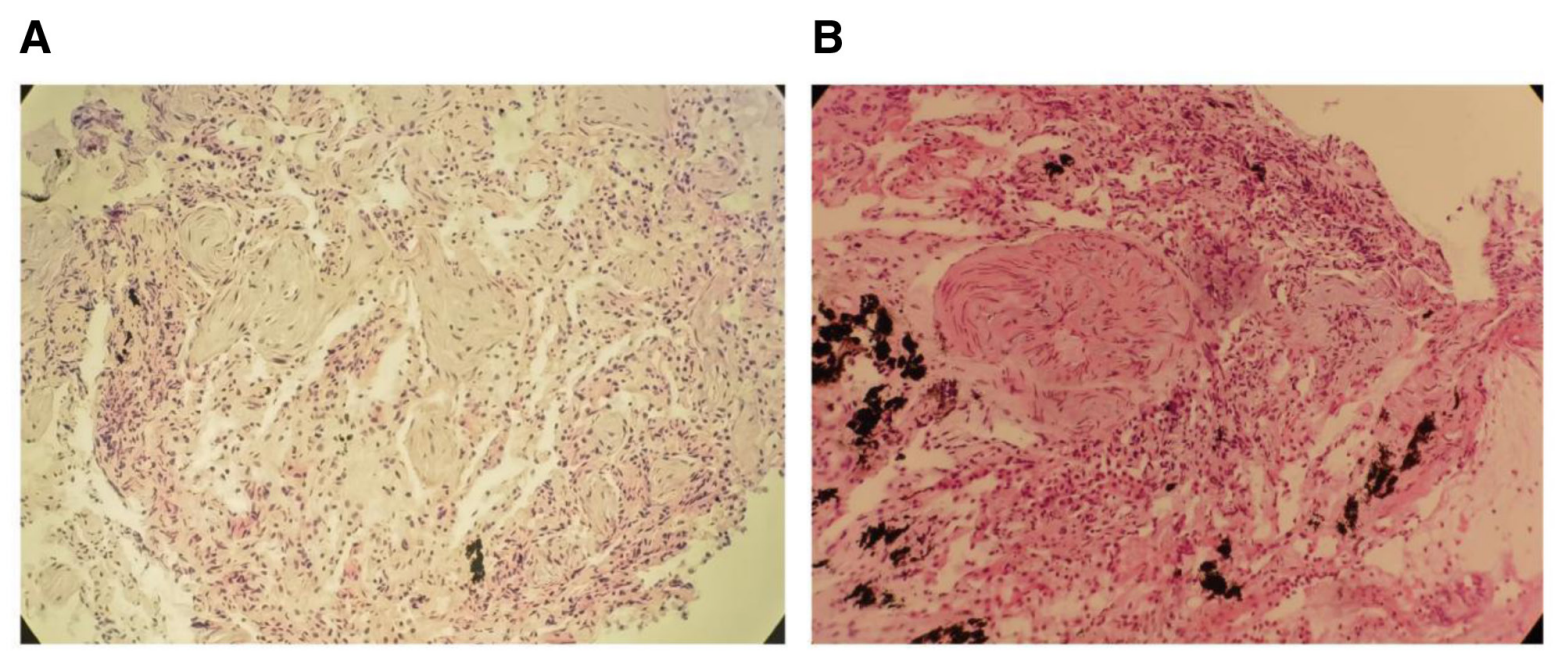
(A) Pathological image of the right lung from the initial diagnosis of organizing pneumonia (H&E, ×200). It revealed fibrinous exudation and organization in segments of the alveolar space, widened alveolar septum, dilated and congested vessels in the alveolar wall, mild hyperplasia of the interstitial fibrous tissue, and infiltration of inflammatory cells. (B) Pathological images of the right lung from the second diagnosis of organizing pneumonia (H&E, ×200). H&E = hematoxylin-eosin.

The patient met the criteria for RA diagnosis according to the 2010 American College of Rheumatology/European League Against Rheumatism classification criteria. The Disease Activity Score using 28 joints [DAS28(ESR)] was 6.95. After 2 weeks of treatment for the infection, the patient’s recurrent fever showed no signs of improvement, and high-resolution chest CT imaging demonstrated an extension of inflammation in the right lung (Fig. 1B). Pathological examination of the lung tissue obtained via fiberoptic bronchoscopy confirmed the presence of OP (Fig. 2A). Treatment with methylprednisolone resulted in the normalization of inflammatory markers and improvement in lung inflammation (Fig. 1C). Owing to intolerance to methotrexate, the patient took 20 mg of leflunomide once daily while gradually tapering off methylprednisolone. The course of glucocorticoid therapy lasted nearly 8 months.

Nine years later, the patient was readmitted to the hospital because of fever that had persisted for 3 days, accompanied by a cough, but without joint swelling or pain. Auscultation of both lungs revealed dry and wet rales. Laboratory results showed a significant elevation in acute-phase reactants, with an ESR of 83 mm/h and a CRP level of 156 mg/L. Additionally, the rheumatoid factor was positive at a high titer of 140.68 IU/mL, whereas the anti-CCP antibody was positive at a low titer of 8.8 U/mL. Serological and sputum tests for viruses, fungi, and tuberculosis yielded negative results. Chest CT revealed bilateral pneumonia with localized bronchiectasis in the middle and lower lobes of the right lung (Fig. 1D, G).

### 2.2. Diagnostic assessment

Pulmonary lesions are thought to result from either a pulmonary infection or pulmonary complications stemming from RA. DAS28(ESR) was 3.2, indicating relatively low disease activity. Following a nearly 2-week course of moxifloxacin as anti-infection therapy, the patient’s fever persisted (Fig. 1E, H). Fiberoptic bronchoscopy was conducted to characterize the lung lesions, including the analysis of bronchoalveolar lavage fluid, detection of a broad spectrum of pathogens, and histopathological assessment of lung tissue from both lobes. Histopathological examination confirmed the diagnosis of OP (Fig. 2B).

### 2.3. Therapeutic intervention

The initial treatment consisted of 40 mg of methylprednisolone administered once daily, followed by gradual tapering. Based on its efficacy in RA and IP, the patient was prescribed 25 mg Iguratimod twice daily for the treatment of rheumatism.

### 2.4. Outcomes

The patient’s fever and cough resolved. Regular chest CT scans showed that lung inflammation improved over time (Fig. 1F, I). The ESR and CRP levels returned to within normal ranges, and the patient discontinued the use of hormones and currently receives treatment with oral iguratimod 25 mg twice daily. Regular follow-ups are being conducted, and the patient reports a high level of satisfaction with the therapeutic outcomes.

## 3. Discussion

UIP and NSIP are relatively common patterns of IP associated with RA, whereas OP is rare. This case stands out because of its unique dual presentation: the concomitant onset of OP and RA, coupled with the relapse of OP. Initially, the patient’s RA was active, as evidenced by the elevated DAS28(ESR). Treatment with methylprednisolone and leflunomide resulted in clinical improvement. After a prolonged course of oral antirheumatic therapy, the patient experienced a more extensive recurrence of pulmonary inflammation 9 years later. Despite the low disease activity suggested by the DAS28-ESR score, the inflammation did not respond to antibiotic therapy, ultimately confirming the diagnosis of OP. OP can appear as the initial presentation of RA or concurrently with or following an RA diagnosis, with the latter being the most common.^[3-5]^ Several patients with a history of smoking or preexisting lung conditions have a notably increased susceptibility to bronchiectasis, particularly among individuals with RA or those with RA compounded by bronchiectasis.^[6]^ The prognosis for such individuals is generally poorer than that for patients without bronchiectasis or those with bronchiectasis alone.^[7,8]^ The patient in this case had a history of bronchiectasis, which is believed to be one of the reasons for OP recurrence.

Glucocorticoids remain the first-line treatment for OP, with a typical dose of 0.5 to 1 mg/kg/d of prednisolone acetate. Previous studies have shown that the average time from glucocorticoid treatment initiation to discontinuation was 180 days.^[4]^ Tapered or abruptly discontinued glucocorticoid therapy increased the risk of relapse. Antirheumatic drugs are often added as adjuncts to glucocorticoids to reduce the risk of recurrence and control disease activity. However, patients with RA who have low disease activity do not require very intensive antirheumatic treatment. In this case, the OP resolved following treatment with an initial dose of 40 mg methylprednisolone, and the disease remained at a low level. Given its mild antirheumatic and bone-protective properties, iguratimod was used for subsequent treatments, and no adverse effects occurred.

A comprehensive study involving 47 patients with connective tissue disease-associated organizing pneumonia (CTD-OP) identified 2 key independent risk factors for recurrence: partial remission following corticosteroid therapy and younger age at OP diagnosis. Furthermore, the presence of residual lesions detected on high-resolution CT scans after treatment with CTD-OP, as well as the occurrence of OP prior to the diagnosis of CTD, were associated with an increased likelihood of OP recurrence.^[9]^ Another study involving 131 patients with cryptogenic and CTD-OP demonstrated that >10% consolidation on HRCT, the presence of detectable bronchiectasis, and a diagnosis of CTD-OP were associated with an increased risk of residual disease.^[10]^

The development of RA-associated IP may be linked to the administration of antirheumatic medications or biologics. A retrospective analysis showed that methotrexate-induced lung disease predominantly arises within the first year of therapy,^[11]^ whereas leflunomide-induced pulmonary fibrosis tends to manifest approximately 20 weeks posttreatment initiation, often in the form of NSIP.^[12]^ There are also scattered reports of drug-induced IP associated with TNF-α inhibitors.^[1,13]^ In the present case, given the timing of antirheumatic drug initiation, the likelihood of drug-induced interstitial lung disease appears to be reduced. We selected iguratimod as an antirheumatic agent based on its efficacy in RA and IP, where patients demonstrated good tolerability and safety.^[14,15]^

OP often presents with fever and cough as initial symptoms, and differentiating between IP and pulmonary infections poses a significant challenge to clinicians. Although various studies have investigated potential biomarkers for RA-associated IP, including autoantibodies, genetic markers, and other markers such as Krebs von den Lungen-6, Matrix Metalloproteinase7, C-X-C motif chemokine ligand 10, and interleukin 18,^[16-18]^ no single biomarker has emerged as a definitive tool for distinguishing infection from other pulmonary lesions. In the present case, the diagnosis was confirmed by pulmonary pathology.

## 4. Conclusion

OP may co-occur with RA, either concurrently or sequentially, and it is more commonly observed during periods of low disease activity. At present, no distinct biomarkers have been established for the diagnosis of OP, and the diagnosis is primarily based on pulmonary tissue examination and the meticulous exclusion of infectious causes. RA-associated OP has a notable recurrence rate and thus requires careful management and vigilant monitoring in clinical practice.

## Author contributions

**Funding acquisition:** Xuebin Wang.

**Investigation:** Fangfang Wang, Fengmei Ge.

**Resources:** Fangfang Wang.

**Supervision:** Xuebin Wang.

**Writing – original draft:** Fangfang Wang.

**Writing – review & editing:** Fangfang Wang, Fengmei Ge.
